# Foreign Body in the Glottis

**DOI:** 10.7759/cureus.24428

**Published:** 2022-04-23

**Authors:** Shalini Shah, Justin Howard, Nutan Winston

**Affiliations:** 1 Anesthesiology, Riverside Community Hospital, Riverside, USA

**Keywords:** airway management, ent procedures, academic anesthesiology, airway foreign body, tracheal foreign body

## Abstract

This case report highlights some of the anesthetic challenges of an airway foreign body removal. We present a case report of foreign body removal in a 50-year-old male with an oxtail bone lodged between the vocal cords. We used face mask general anesthesia with sevoflurane.

## Introduction

Foreign body aspiration is an uncommon but potentially life-threatening event in adults, accounting for 0.16-0.33% of adult bronchoscopy procedures. While the majority of accidental aspiration occurs in children, adults represent up to 25% of cases [1]. Common aspirated objects include food and broken prices of dental appliances [2]. Diagnosis of foreign body aspiration can be confirmed with imaging. A variety of sedation approaches are possible, including conscious sedation with spontaneous ventilation, general anesthesia with laryngeal mask airway or endotracheal tube, and rigid bronchoscopy with jet ventilation. This case report highlights the anesthesia management.

## Case presentation

**Figure 1:**
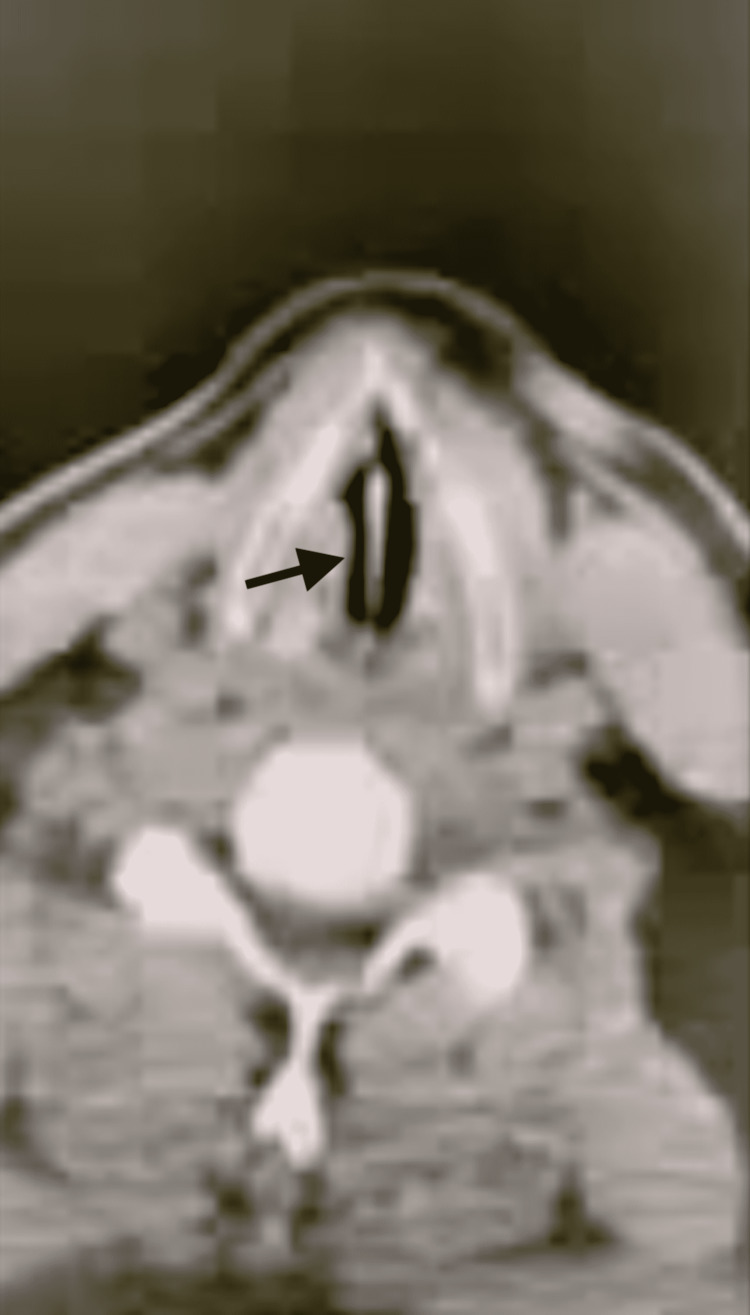
CT of the foreign body in the airway

A 50-year-old male with a past medical history of diabetes mellitus and tobacco abuse presented to the hospital after eating oxtail (Figure 1). The patient reported odynophagia, hemoptysis, and an inability to clear his throat. Even though the patient was not overly distressed, foreign body aspiration was suspected. Vital signs on admission were as follows: heart rate was 79 bpm, blood pressure was 136/74 mmHg, SpO2 was 97, and respiration rate was 20 breaths per minute. No respiratory distress, stridor, or labored breathing was noted upon examination. A 3-cm lesion in the larynx was seen on a radiograph of the neck. A computer tomography (CT) image of the foreign body seen between the vocal cords is provided in Figure 1. Intraoperative management included adequate preoxygenation. The patient was mask-induced with sevoflurane while maintaining spontaneous respiration. To achieve a deep level of anesthesia, intermittent boluses of propofol and fentanyl were used during induction. After achieving adequate depth of anesthesia, the surgeon performed direct laryngoscopy, followed by rigid bronchoscopy to remove the foreign body. During the case, the patient started moving and became tachycardic and hypertensive. An insufficient depth of anesthesia was suspected, and mask ventilation was resumed with sevoflurane. Dexamethasone 10 mg was given after the foreign body was removed to treat moderate vocal cord swelling. Postoperatively, the patient received racemic epinephrine, along with a cool mist mask to further decrease vocal cord swelling.

## Discussion

Foreign body aspiration is an emergent situation that can occasionally occur in adults. Patients can be asymptomatic or present with sudden and persistent coughing, dyspnea, wheezing, and stridor. Foreign body aspiration can cause hypoxemia and respiratory arrest if the obstruction is severe. Common causes of foreign body aspiration in adults are food particles or broken parts of dentures. The foreign body can be lodged in any portion of the airway depending on the size and shape of the object. Diagnosis of foreign body aspiration can be confirmed with imaging. Bronchoscopy with foreign body removal under anesthesia is the standard therapy for foreign body aspiration removal [3,4]. This procedure can be difficult due to sharing of the airway between surgery and anesthesia. Currently, intravenous and/or inhalational anesthesia with spontaneous breathing is the preferred method of anesthesia technique during this procedure [5].

Our anesthetic management consisted of general anesthesia using intermittent propofol and sevoflurane. First, a face mask with 10 liters of 100% oxygen was used to ensure adequate oxygenation reserve. Anesthetic induction was then achieved with propofol. Sevoflurane was used to briefly deepen the anesthesia via the face mask enough for the surgeon to perform the bronchoscopy while still maintaining spontaneous ventilation.

Even though the foreign body removal operation duration was short, there were difficulties in anesthetic management. The difficulties mainly came from field avoidance. When the surgeon initially performed the rigid bronchoscopy for foreign body removal from the trachea, the patient became tachycardic and hypertensive and started moving during attempted extraction. This indicated that the anesthetic depth was inadequate. We then administered an additional bolus of propofol and fentanyl and took over the airway to provide oxygenation and sevoflurane to achieve an adequate level of anesthesia. The surgeon was then allowed to resume the operation. The next issue came from desaturation during extraction. Due to the size and shape of the foreign body, the surgeon needed to delicately remove the object while preventing additional airway trauma or causing a portion of the foreign body to break and travel further into the airway. During this time, the patient’s oxygen saturation started to drop with spontaneous ventilation. When the saturation dropped to 92%, we told the surgeon we needed to oxygenate the patient before continuing any further in the operation. The patient was oxygenated with 10 liters of oxygen via the face mask. We could not use positive pressure ventilation to prevent the foreign body from potentially traveling further into the airway [5]. When adequate oxygenation and anesthetic depth was achieved again, the surgeon was allowed to continue. After removal of the foreign body, the vocal folds were found to be edematous and inflamed. The patient was given 10 mg of intravenous dexamethasone prior to leaving the operating room to decrease vocal cord swelling. In the postanesthesia care unit, the patient was given humidified oxygen and three doses of racemic epinephrine. He was discharged home uneventfully once postoperative milestones were met.

## Conclusions

There are many reported cases of foreign body aspiration into the trachea, bronchial tree, and other locations in the airway. We presented a case of foreign body aspiration of a large object in an adult and explored the anesthetic management and challenges inherently found with foreign body removal in the upper airway. Monitored anesthesia care with spontaneous ventilation is the preferred method of anesthetic management. However, this method of anesthesia and adequate oxygenation can be difficult to maintain due to sharing the airway with the surgical team. Proper communication between surgery and anesthesia teams must occur for patient safety during this operation.
